# Rapid Diagnosis of *Pneumocystis jirovecii* Pneumonia and Respiratory Tract Colonization by Next-Generation Sequencing

**DOI:** 10.1007/s11046-024-00849-y

**Published:** 2024-05-05

**Authors:** Fanfan Xing, Chaowen Deng, Zhendong Luo, Shan Zou, Min Liu, Haiyan Ye, Linlin Sun, Chi-Ching Tsang, Simon K. F. Lo, Susanna K. P. Lau, Patrick C. Y. Woo

**Affiliations:** 1 Department of Clinical Microbiology and Infection Control, The University of Hong Kong–Shenzhen Hospital, Shenzhen, Guangdong China; 2 Department of Radiology, The University of Hong Kong–Shenzhen Hospital, Shenzhen, Guangdong China; 3 Department of Adult Intensive Care, The University of Hong Kong–Shenzhen Hospital, Shenzhen, Guangdong China; 4 Department of Respiratory Medicine, The University of Hong Kong–Shenzhen Hospital, Shenzhen, Guangdong China; 5 School of Medical and Health Sciences, Tung Wah College, Homantin, Hong Kong China; 6 Department of Microbiology, School of Clinical Medicine, Li Ka Shing Faculty of Medicine, The University of Hong Kong, Hong Kong, China; 7 Doctoral Program in Translational Medicine and Department of Life Sciences, National Chung Hsing University, Taichung 402, Taiwan; 8 The iEGG and Animal Biotechnology Research Center, National Chung Hsing University, Taichung 402, Taiwan

**Keywords:** *Pneumocystis jirovecii*, Pneumonia, Respiratory tract, Colonization, Next-generation sequencing

## Abstract

**Objectives:**

To describe the epidemiology of *Pneumocystis jirovecii* pneumonia and colonization diagnosed by next-generation sequencing (NGS) and explore the usefulness of the number of *P. jirovecii* sequence reads for the diagnosis of *P. jirovecii* pneumonia.

**Methods:**

We examined the NGS results for *P. jirovecii* in respiratory samples collected from patients and analysed their clinical, radiological and microbiological characteristics.

**Results:**

Among 285 respiratory samples collected over a 12-month period (January to December 2022), *P. jirovecii* sequences were detected in 56 samples from 53 patients. Fifty (94.3%) of the 53 patients were HIV-negative. Following our case definitions, 37 (69.8%) and 16 (30.2%) of the 53 patients had *P. jirovecii* infection and colonization respectively. *P. jirovecii* infection was associated with presence of underlying disease with immunosuppression (94.6% vs 18.8%, *P* < 0.05), positive serum 1,3-β-D-glucan (41.2% vs 0%, *P* < 0.01) and higher number of *P. jirovecii* sequence reads (*P* < 0.005). In contrast, *P. jirovecii* colonization was associated with the male sex (93.8% vs 54.1%, *P* < 0.01), another definitive infectious disease diagnosis of the respiratory tract (43.8% vs 2.7%, *P* < 0.001) and higher survival (100% vs 67.6%, *P* < 0.01). Although *P. jirovecii* pneumonia was associated with higher number of *P. jirovecii* reads in respiratory samples, only a sensitivity of 82.14% and a specificity of 68.75% could be achieved.

**Conclusion:**

Detection of *P. jirovecii* sequences in respiratory samples has to be interpreted discreetly. A combination of clinical, radiological and laboratory findings is still the most crucial in determining whether a particular case is genuine *P. jirovecii* pneumonia.

**Supplementary Information:**

The online version contains supplementary material available at 10.1007/s11046-024-00849-y.

## Introduction

*Pneumocystis jirovecii* is a fungus that causes pneumonia in immunocompromised patients. Clinically *P. jirovecii* pneumonia is characteristically associated with fever, shortness of breath and hypoxia and radiologically ground glass opacities are often observed. In general, it causes a relatively milder disease with lower (10–12%) mortality in human immunodeficiency virus (HIV)-positive patients but more severe disease with higher (30–50%) mortality in other immunocompromised patients who are HIV-negative [1]. Traditionally, laboratory diagnosis of *P. jirovecii* pneumonia was achieved by direct detection of *P. jirovecii* asci in respiratory tract specimens by microscopic examination after Grocott-Gomori methenamine silver (GMS) or immunofluorescence staining [2]. In recent years, polymerase chain reaction (PCR) has also been used for the detection of *P. jirovecii* [3–6]. Although it has improved the sensitivity of detection, it is not able to distinguish between genuine *P. jirovecii* pneumonia and *P. jirovecii* colonization of the respiratory tract [7, 8].

In the last few years, next-generation sequencing (NGS) has emerged as a technology for laboratory diagnosis of many culture-negative infections [9, 10]. We have recently reported its application in confirming the first case of listeria meningitis in a patient with autoantibody against interferon gamma as well as understanding the spectrum of Q fever, fungal infections and culture-negative meningitis and encephalitis [10–13]. It is notable that we have shown, in our recent review, that in fact *P. jirovecii* is the commonest fungal organism detected by NGS in clinical specimens [10]. Furthermore, others have also shown that NGS is much more sensitive than direct GMS staining and microscopy for the detection of *P. jirovecii* in respiratory samples [14–16]. In this study, we describe the epidemiology of *P. jirovecii* infection and colonization diagnosed by NGS in our hospital and discuss the reasons that may account for such phenomena. In addition, we also explore the usefulness of the number of *P. jirovecii* sequence reads for the diagnosis of *P. jirovecii* pneumonia and discuss how to interpret NGS results.

## Materials and Methods

### Ethical Statement

This study was approved by the Institutional Review Board of The University of Hong Kong—Shenzhen Hospital ([2022]120), and the requirement of obtaining informed consent was exempted.

### Patients

This study was conducted over a 12-month period (January to December 2022) in The University of Hong Kong—Shenzhen Hospital, Shenzhen, China. This 1,400-bed multi-specialty hospital was established in 2012 and provides primary to tertiary medical services to the residents of Shenzhen city in both inpatient and outpatient settings. Supported through the policy from the government of Shenzhen, the hospital is established as a reform model medical institution in China, and many new medical technologies can be introduced to the hospital first. The laboratory reports of all respiratory samples submitted for NGS were examined. The clinical details, laboratory data and radiological findings of all patients with *P. jirovecii* sequence reads detected in their respiratory samples were retrieved from the hospital electronic record system and analysed.

### Case Definitions

According to the Consensus Definitions of Invasive Fungal Disease of the European Organization for Research and Treatment of Cancer and the Mycoses Study Group [17], a case of *P. jirovecii* infection is defined as a *P. jirovecii* NGS-positive patient, whom after careful consideration of his/her clinical, radiological and laboratory findings, the clinician-in-charge has decided to prescribe specific anti-*P. jirovecii* treatment. A case of *P. jirovecii* colonization is defined as a *P. jirovecii* NGS-positive patient, whom after careful consideration of his/her clinical, radiological and laboratory findings, the clinician-in-charge has decided not to prescribe specific anti-*P. jirovecii* treatment.

### Microbiological and Other Laboratory Tests

Clinical specimens were collected and handled according to standard protocols [18]. Direct detection of *P. jirovecii* and acid-fast bacilli were performed by GMS stain and Ziehl–Neelsen stain, respectively. The identities of bacterial and fungal isolates were confirmed by matrix-assisted laser desorption ionization–time-of-flight mass spectrometry [19]. Cryptococcal antigen detection was performed using lateral flow assay (Norman, USA). 1,3-β-D-glucan detection was performed using Test Kit for the Detection of Fungus 1,3-β-D-Glucan (Photometric Assay) (A & C Biological Ltd, Zhanjiang, China). Real-time PCR for *Mycobacterium tuberculosis* was performed using *M. tuberculosis* DNA Fluorescence Diagnostic Kit (PCR-Fluorescence Probing) (Sansure Biotech, Hunan, China); and real-time RT-PCR for severe acute respiratory syndrome coronavirus 2 (SARS-CoV-2) was performed using 2019-nCoV Nucleic Acid Test Kit (Biogerm, Shanghai, China). Real-time RT-PCR for influenza virus A and real-time PCR for herpes simplex virus (HSV) were performed by KingMed Diagnostics company.

### NGS

The sputum and bronchoalveolar lavage (BAL) samples were sent to KingMed Diagnostics company, Sagene company, Vision Medicals company, Dinfectome company or GensKey company for targeted NGS (tNGS) or metagenomics NGS (mNGS) analysis.

### Statistical Analysis

A comparison of characteristics between the *P. jirovecii* infection and colonization groups was performed. Chi-square test was used for categorical variables and unpaired Student’s t-test or Mann–Whitney U test was used for continuous variables. *P* < 0.05 was considered as statistically significant.

## Results

### Patients

During the 12-month study period, a total of 285 respiratory samples from 241 patients were submitted for tNGS or mNGS analyses. Among these 285 samples, *P. jirovecii* sequence reads were detected in 56 samples from 53 patients. For these 53 patients, the male to female ratio was 35:18. The median age was 61 (range 30 to 85) years. Thirty-eight (71.7%) of the 53 patients had underlying immunocompromised conditions, the commonest being malignancies (n = 19), followed by connective tissue and autoimmune diseases on corticosteroid and/or other immunosuppressive treatment (n = 15), solid organ transplant recipients on corticosteroid and/or other immunosuppressive treatment (n = 4) and HIV infection (n = 3) (Table 1).

**Table 1:** Demographic and clinical characteristics of patients in whom *Pneumocystis jirovecii* was detected by NGS

Case no	Sex/age	Underlying disease(s)	Key clinical manifestation(s)	Immunosuppressive and/or chemotherapy of underlying disease(s)	Colonization/infection of *P. jirovecii*	Anti-*P. jirovecii* treatment	Outcome
1	F/51	Dermatomyositis, ILD	Fever, skin rash and rupture, SOB	Methylprednisolone, hydroxychloroquine, tacrolimus	Infection	TMP-SMX	Improved
2	M/72	Renal transplantation, DM	Cough, fever	Cyclosporin A, prednisone, MMF	Colonization	None	Improved
3	M/44	Renal transplantation	Cough, SOB, fever	Tacrolimus, prednisone	Infection	TMP-SMX	Improved
4	M/66	Hepatocellular carcinoma, chronic HBV infection, immune pneumonitis	Fever, diarrhea, SOB	Prednisone, MMF, sorafenib, camrelizumab, apatinib, radiotherapy	Infection	TMP-SMX	Succumbed
5	F/69	Brest carcinoma	SOB	Abemaciclib	Infection	TMP-SMX	Succumbed
6	M/67	Lung transplantation, DM, COPD, pulmonary heart disease, CKD	SOB	Tacrolimus, prednisone, MMF	Infection	TMP-SMX, caspofungin, clindamycin	Succumbed
7	F/45	Breast carcinoma	SOB	Doxorubicin, cyclophosphamide, paclitaxel, dexamethasone	Infection	TMP-SMX	Improved
8	M/40	Hyperthyroidism, gout	Fever, headache	None	Colonization	None	Improved
9	F/49	Neuromyelitis optica spectrum disorders, Hashimoto's thyroiditis	Fever, headache, limb spasticity, SOB	MMF, prednisone	Infection	TMP-SMX	Improved
10	F/48	Breast carcinoma, adult Still's disease	SOB, fever	Radiotherapy, methylprednisolone, cyclosporin A	Infection	TMP-SMX	Improved
11	M/58	MDS	Cough, SOB	None	Infection	TMP-SMX	Improved
12	M/47	Chronic HBV infection, AIDS	Cough, fatigue, fever	None	Infection	TMP-SMX	Improved
13	M/51	Nasopharyngeal carcinoma, renal transplantation, chronic HBV infection	Facial edema, SOB, cough, sore throat	Tacrolimus, MMF, paclitaxel, cisplatin, capecitabine, tegafur, gimeracil, oteracil, gemcitabine, cetuximab, docetaxel, nimotuzumab, radiotherapy, anlotinib	Infection	Caspofungin, clindamycin	Succumbed
14	M/67	Hypertension, antisynthetase syndrome	Cough, fever	None	Infection	TMP-SMX	Improved
15	M/47	Chronic HBV infection, bronchiectasis, hamartoma of left lung	Cough, fever	None	Colonization	None	Improved
16	M/54	Meningioma, ANCA-associated small-vessel vasculitis, COPD	Cough	None	Infection	TMP-SMX	Improved
17	F/69	Lung carcinoma	Fever, SOB	Almonertinib, osimertinib, dexamethasone	Infection	TMP-SMX	Improved
18	F/80	RA, ILD, pulmonary arterial hypertension	SOB, fever	Leflunomide, hydroxychloroquine, prednisone, dexamethasone, iguratimod, tripterygium glycosides, tofacitinib, denosumab	Infection	TMP-SMX	Improved
19	M/63	COPD	SOB, cough, hemoptysis	None	Colonization	None	Improved
20	F/66	Overlap syndrome	Cough, recurrent SOB	Prednisone, hydroxychloroquine, azathioprine	Colonization	None	Improved
21	M/71	Membranous nephropathy, DM, hypertension, CHD	General edema	None	Infection	TMP-SMX	Improved
22	F/35	Myasthenia gravis, Ekbom syndrome, xerophthalmia, post-resection of thymoma	SOB	Prednisone, azathioprine	Infection	TMP-SMX, caspofungin, clindamycin	Improved
23	M/62	Asthma, ABPA	SOB, fever	None	Colonization	None	Improved
24	M/82	CKD	Cough, SOB	None	Colonization	None	Improved
25	F/59	Breast carcinoma, radiation pneumonitis	Cough, SOB	Pharmorubicin, cyclophosphamide, docetaxel, abemaciclib	Infection	TMP-SMX	Improved
26	M/42	Aplastic anemia, community acquired pneumonia	Cough, sore throat, fever	None	Colonization	None	Improved
27	M/63	Subacute combined degeneration	Fatigue, myalgia	Methylprednisolone	Infection	TMP-SMX	Improved
28	F/53	T cell lymphoma	SOB	None	Infection	TMP-SMX	Improved
29	F/52	Breast carcinoma	None	Doxorubicin, cyclophosphamide, paclitaxel, dexamethasone	Infection	TMP-SMX	Improved
30	F/46	Breast carcinoma	Fever, cough	Doxorubicin, cyclophosphamide, paclitaxel, dexamethasone	Infection	TMP-SMX	Improved
31	M/61	Mantle cell lymphoma	Erythema, desquamation, fever	Zanubrutinib, prednisone	Infection	TMP-SMX	Improved
32	M/52	Chronic HBV infection, mediastinal solitary fibrous tumor	Fever, cough	None	Colonization	None	Improved
33	M/46	Thymoma, myasthenia gravis, bronchiectasis	Cough	Methylprednisolone	Infection	TMP-SMX, caspofungin, clindamycin	Improved
34	M/73	T cell lymphoma, DM, liver cirrhosis	Cough, SOB, fever	Chidamide, thalidomide, lenalidomide, cisplatin, gemcitabine, L-asparaginase, ifosfamide, etoposide, vincristine, dexamethasone, pomalidomide, cytarabine	Infection	TMP-SMX	Succumbed
35	M/75	Gastric carcinoma	None	Oxaliplatin, capecitabine	Infection	None	Succumbed
36	M/65	ILD	Palpitation, cough, hemoptysis, chest distress	Prednisone, nintendanib, methylprednisolone, pirfenidone	Infection	TMP-SMX	Succumbed
37	F/36	Breast carcinoma	Chest distress, SOB	Doxorubicin, cyclophosphamide, paclitaxel	Infection	TMP-SMX	Improved
38 [20]	F/71	Hemophagocytic lymphohistiocytosis, Still's disease	Fever, fatigue, chest distress, SOB	Dexamethasone, prednisone, cyclosporine, tocilizumab	Infection	TMP-SMX	Succumbed
39	M/66	DM	Fever	None	Colonization	None	Improved
40	F/55	Anti-MDA5 antibody dermatomyositis	Cough, palpitation	None	Infection	TMP-SMX	Succumbed
41	M/30	AIDS	Fever, SOB	None	Infection	TMP-SMX	Improved
42	F/55	Breast carcinoma	Fever	Doxorubicin, cyclophosphamide	Infection	TMP-SMX	Improved
43	M/85	DM	Fever, cough	None	Colonization	None	Improved
44	M/73	COPD, CHD, gout, hypertension, renal calculi, BPH	Fever, cough, coma	None	Infection	TMP-SMX	Succumbed
45	M/44	AIDS	SOB, cough, diarrhea	None	Infection	TMP-SMX	Improved
46	M/66	DM	None	None	Colonization	None	Improved
47	M/66	None	Cough, fever	None	Colonization	None	Improved
48	M/84	DM	Cough, sputum	None	Colonization	None	Improved
49	F/75	Ovarian malignant teratoma, RA	SOB, fever	Bleomycin, etoposide, cisplatin	Infection	TMP-SMX	Succumbed
50	M/67	COPD	Fever, cough	None	Colonization	None	Improved
51	M/55	None	SOB, cough, fever	None	Colonization	None	Improved
52	M/73	RA, ILD	SOB, fever	Methotrexate, hydroxychloroquine, sulfasalazine, iguratimod	Infection	TMP-SMX	Improved
53	M/53	Dermatomyositis, ILD	SOB, cough	Prednisone, cyclophosphamide, pirfenidone	Infection	TMP-SMX	Succumbed

F, Female; M, Male; ILD, Interstitial lung disease; SOB, Shortness of breath; TMP-SMX, Trimethoprim-sulfamethoxazole; DM, Diabetes mellitus; MMF, Mycophenolate mofetil; HBV, Hepatitis B virus; COPD, Chronic obstructive pulmonary disease; CKD, Chronic kidney disease; MDS, Myelodysplastic syndrome; AIDS, Acquired immune deficiency syndrome; ANCA, Anti-neutrophil cytoplasmic antibodies; RA, Rheumatoid arthritis; CHD, Coronary heart disease; ABPA, Allergic bronchopulmonary aspergillosis; MDA5, Melanoma differentiation-associated protein 5; BPH, Benign prostatic hyperplasia

### NGS Analysis

In 44 of the 53 patients, *P. jirovecii* was detected by tNGS; whereas in nine patients, it was detected by mNGS. In samples collected from 31 (58.5%) of the 53 patients, sequence reads of other potential respiratory pathogens were detected (Table 2). These included bacteria (*Acinetobacter baumannii*, *Bordetella pertussis, Chlamydia psittaci*, *Haemophilus influenzae*, *Klebsiella pneumoniae*, *Legionella pneumophilia*, *Pseudomonas aeruginosa*, *Staphylococcus aureus*, *Stenotrophomonas maltophilia* and *Streptococcus pneumoniae*), mycobacteria (*Mycobacterium abscessus*, *Mycobacterium chelonae*, *Mycobacterium intracellulare*, *Mycobacterium kansasii* and *M. tuberculosis*), viruses (adenovirus, influenza virus and rhinovirus) and fungi (*Aspergillus fumigatus*, *Cryptococcus neoformans* and *Trichosporon asahii*). In samples collected from 26 (49.1%) of the 53 patients, sequences that were considered as contaminants or colonizers were present. Most of them were bacteria and yeasts present in the oral cavity of immunocompetent or immunocompromised hosts (Supplementary Table 1). In samples collected from eight (cases 5, 17, 29, 30, 31, 32, 37 and 51) of the 53 patients, *Tropheryma whipplei*, a bacterium of doubtful clinical significance in the respiratory tract [21], was detected (Table 2).

**Table 2:** NGS analysis and other key laboratory results of patients in the present cohort

Case no	Gomori methenamine silver staining	NGS			1,3-β-D-glucan (pg/mL)	Other positive microbiological tests
		Specimen	tNGS/mNGS	Sequencing result (number of reads)		
1	Negative	BAL	mNGS	*Pneumocystis jirovecii* (5), *Prevotella melaninogenica* (81), *Veillonella parvula* (5), *Mycobacterium intracellulare* (1)	201.61	None
2	Not done	Sputum	tNGS	*Enterobacter cloacae* complex (9), *P. jirovecii* (19), EBV (17,782), CMV (50), HHV-7 (49), *Ureaplasma urealyticum* (2)	< 37.5	None
3	Negative	BAL	tNGS	*P. jirovecii* (1886)	< 37.5	None
4	Negative	BAL	mNGS	*Rothia mucilaginosa* (45,448), *Streptococcus mitis* (13,051), *Streptococcus pneumoniae* (11,961), *P. jirovecii* (168), HSV-1 (17), *Olsenella uli* (18,072), *P. melaninogenica* (8702), *Staphylococcus haemolyticus* (6259), *V. parvula* (3530), *Parvimonas micra* (3037), *Corynebacterium simulans* (1984), *Cryptobacterium curtum* (1390), *Filifactor alocis* (379), *Leptotrichia buccalis* (329), *Corynebacterium striatum* (269), *Atopobium parvulum* (261), *Peptostreptococcus anaerobius* (152), *Clostridioides difficile* (136), *Actinomyces oris* (82)	> 600	None
5	Negative	Sputum	tNGS	*Tropheryma whipplei* (19,095), *Haemophilus influenzae* (387), *Staphylococcus aureus* (26), *P. jirovecii* (14), rhinovirus C (16,789), EBV (7569)	< 37.5	None
6	Negative	BAL	tNGS	*Candida albicans* (27,428), EBV (3357), *Enterococcus faecalis* (1925), *Stenotrophomonas maltophilia* (321), *P. jirovecii* (141), HHV-7 (136), *Trichosporon asahii* (34)	< 37.5	None
7	Negative	BAL	mNGS	*S. aureus* (3), *P. jirovecii* (2), *Neisseria flavescens* (40), *Haemophilus parainfluenzae* (15), *P. melaninogenica* (15), *R. mucilaginosa* (12), *Porphyromonas gingivalis* (11), *Fusobacterium nucleatum* (6), *Capnocytophaga granulosa* (4), *Peptostreptococcus stomatis* (4), *Aggregatibacter segnis* (4), *F. alocis* (4), *Treponema denticola* (4), *Veillonella dispar* (4), *Streptococcus pseudopneumoniae* (3)	< 37.5	None
8	Not done	Sputum	tNGS	*Chlamydia psittaci* (49), *Acinetobacter baumannii* (177), *H. influenzae* (33), *Pseudomonas aeruginosa* (22), *S. maltophilia* (14), HHV-7 (32), *P. jirovecii* (11), HHV-6 (6)	Not done	None
9	Not done	BAL	mNGS	*P. jirovecii* (830), *Streptococcus* species (23), *R. mucilaginosa* (10), *Abiotrophia defectiva* (7), *Granulicatella adiacens* (5), *Staphylococcus epidermidis* (3), *Tannerella forsythia* (2), *Prevotella denticola* (1), EBV (1), Torque teno virus (1)	> 600	None
10	Positive	BAL	tNGS	*P. jirovecii* (48,609), EBV (3)	394.01	None
11	Negative	BAL	mNGS	*S. aureus* (79), *C. albicans* (1757), *P. jirovecii* (2), human polyomavirus 5 (4), *Mycobacterium chelonae* (3), *S. haemolyticus* (264,865), *Lactobacillus rhamnosus* (12,454), *Mogibacterium timidum* (72), *Corynebacterium tuberculostearicum* (9)	< 37.5	None
12	Positive	BAL	tNGS	*P. jirovecii* (84,000), *Bordetella pertussis* (1705), rhinovirus A (44)	49.72	None
13	Not done	Sputum	tNGS	*Klebsiella pneumoniae* (44,975), *A. baumannii* (15,986), *S. maltophilia* (11,464), *E. faecalis* (11,480), EBV (826), *P. jirovecii* (87), *E. cloacae* complex (32), HSV-1 (9)	< 37.5	None
14	Not done	BAL	mNGS	*P. jirovecii* (15), *P. melaninogenica* (1405), *R. mucilaginosa* (930), *Campylobacter concisus* (432), *Streptococcus infantis* (363), *Gemella sanguinis* (269), *Veillonella atypica* (189), *Eikenella corrodens* (123), *Actinomyces graevenitzii* (115), *Solobacterium moorei* (104), *Capnocytophaga sputigena* (101), *H. parainfluenzae* (83), *A. defectiva* (66), *L. buccalis* (54), *P. stomatis* (51), *Oribacterium sinus* (45)	< 37.5	None
15	Negative	Sputum	tNGS	EBV (1215), HHV-7 (741), *P. jirovecii* (72)	< 37.5	None
16	Negative	BAL	tNGS	*P. jirovecii* (4476), *C. albicans* (4447), *Legionella pneumophilia* (17), HHV-7 (83), CMV (5)	< 37.5	None
	Negative	BAL	tNGS	*P. jirovecii* (322), *C. albicans* (178), CMV (12), HHV-7 (10)	< 37.5	None
17	Negative	BAL	tNGS	*C. albicans* (424), *P. jirovecii* (135), CMV (135), *H. influenzae* (19), *T. whipplei* (54), HHV-7 (6)	61.96	None
18	Negative	BAL	tNGS	Rhinovirus (192), *P. aeruginosa* (278), *P. jirovecii* (105), *S. pneumoniae* (5), EBV (7)	183.52	Sputum culture: *P. aeruginosa*
19	Not done	Sputum	tNGS	*P. aeruginosa* (40,038), EBV (882), CMV (23), HHV-7 (12), *P. jirovecii* (7)	< 37.5	Sputum culture: *P. aeruginosa*
20	Not done	Sputum	tNGS	*P. aeruginosa* (48,178), *Mycobacterium kansasii* (150), *S. maltophilia* (603), EBV (274), *P. jirovecii* (166), CMV (115), *C. albicans* (27), HHV-7 (19)	< 37.5	Sputum for AFB smear: positive; sputum culture: *P. aeruginosa*
21	Not done	Sputum	tNGS	*S. aureus* (6472), *P. jirovecii* (184), HHV-7 (136), *Haemophilus haemolyticus* (113)	< 37.5	None
22	Not done	Sputum	tNGS	*Mycobacterium abscessus* (3907), *H. influenzae* (46,294), *H. haemolyticus* (355), *P. jirovecii* (212), EBV (129), HSV-1 (97), CMV (32), HHV-7 (30)	Not done	None
23	Negative	Sputum	tNGS	Influenza virus A (38,696), *Aspergillus fumigatus* (76), *P. jirovecii* (1)	< 37.5	NPS for Influenza A virus RNA: positive
24	Not done	Sputum	tNGS	*K. pneumoniae* (54,414), *A. baumannii* (10,292), *E. faecalis* (8694), EBV (1517), CMV (93), *S. aureus* (88), HHV-7 (46), *P. jirovecii* (13)	Not done	None
25	Negative	BAL	tNGS	*P. jirovecii* (172)	< 37.5	None
26	Not done	Sputum	tNGS	*A. baumannii* (173), *H. haemolyticus* (51), *S. aureus* (21), *P. jirovecii* (8)	< 37.5	None
27	Negative	BAL	mNGS	*Escherichia coli* (61,948), *K. pneumoniae* (1424), *E. cloacae* complex (3289), *P. jirovecii* (1104), HSV (1,095,105), EBV (53), HHV-7 (26), HHV-6B (4), *S. epidermidis* (494,451), *V. parvula* (215,980), *Actinomyces dentalis* (22,892), *S. infantis* (46,135), *Campylobacter curvus* (32,006), *Neisseria bacilliformis* (27,509), *Corynebacterium matruchotii* (12,641), *Cutibacterium acnes* (11,078), *E. corrodens* (12,967), *C. albicans* (4452), *Trichomonas tenax* (723)	128.64	BAL culture: *K. pneumoniae*, *C. albicans*
28	Not done	Sputum	tNGS	*P. jirovecii* (117), HHV-7(11)	< 37.5	None
29	Negative	BAL	tNGS	*P. jirovecii* (4137), *P. aeruginosa* (27), *E. coli* (14), *T. whipplei* (749)	Not done	BAL culture: *P. aeruginosa*
30	Negative	BAL	tNGS	*P. jirovecii* (655), *T. whipplei* (14)	< 37.5	None
31	Negative	BAL	mNGS	*P. jirovecii* (878), *T. whipplei* (234), Torque teno virus (510), CMV (200), *S. pseudopneumoniae* (280), *Streptococcus oralis* (57), *H. parainfluenzae* (73), *H. haemolyticus* (10), *G. adiacens* (14), *R. mucilaginosa* (13)	87.61	None
32	Not done	Sputum	tNGS	*K. pneumoniae* (6055), *H. haemolyticus* (1738), EBV (330), *S. aureus* (94), *T. whipplei* (366), HHV-7 (77), *P. jirovecii* (32), HHV-6 (11), CMV (5)	Not done	None
33	Not done	Sputum	tNGS	*H. haemolyticus* (1960), HHV-7 (365), *P. jirovecii* (274), *T. asahii* (1), EBV (34), *C. albicans* (7)	87.6	None
	Not done	Sputum	tNGS	*H. haemolyticus* (1385), *H. influenzae* (14,635), HHV-7 (548), EBV (77), *P. jirovecii* (45), *C. albicans* (20)		None
	Not done	BAL	tNGS	*P. jirovecii* (189), *C. albicans* (23), CMV (6), HHV-7 (4)		None
34	Not done	Sputum	tNGS	*C. albicans* (20,746), *A. baumannii* (1112), *K. pneumoniae* (340), *P. jirovecii* (260)	76.97	Sputum culture: *A. baumannii*, *C. albicans*
35	Not done	BAL	tNGS	Nontuberculosis mycobacteria (185), *P. jirovecii* (52)	Not done	None
36	Negative	Sputum	tNGS	*K. pneumoniae* (24,020), *H. influenzae* (8038), EBV (199), *C. albicans* (163), *A. baumannii* (41), HHV-7 (36), *P. jirovecii* (27)	< 37.5	BAL culture: *K. pneumoniae*
37	Negative	BAL	tNGS	*P. jirovecii* (9600), *T. whipplei* (8)	225.8	None
38 [20]	Positive	BAL	tNGS	*P. jirovecii* (34,019), CMV (2975), EBV (8), *K. pneumoniae* (21), *C. albicans* (2)	293.99	Blood and pleural effusion culture: *Nocardia kroppenstedtii*; BAL culture: *K. pneumoniae*, *N. kroppenstedtii*
39	Negative	BAL	tNGS	*Mycobacterium tuberculosis* complex (43,834), *P. jirovecii* (927)	< 37.5	BAL for AFB smear: positive; BAL for *M. tuberculosis* DNA: positive
40	Positive	BAL	tNGS	*P. jirovecii* (25,284), EBV (17)	< 37.5	None
41	Positive	BAL	tNGS	*P. jirovecii* (58,385), EBV (374), *E. faecalis* (199)	93.8	None
42	Negative	BAL	tNGS	*P. jirovecii* (2470)	< 37.5	None
43	Negative	BAL	tNGS	HSV-1 (580), *E. coli* (11), HHV-7 (4), *C. albicans* (10,441), *P. jirovecii* (49)	< 37.5	BAL for HSV DNA: positive
44	Negative	BAL	tNGS	*P. aeruginosa* (3638), *K. pneumoniae* (58), CMV (61), *P. jirovecii* (47)	< 37.5	BAL culture: *P. aeruginosa*
45	Positive	BAL	tNGS	*P. jirovecii* (64,629), CMV (81)	< 37.5	None
46	Not done	BAL	tNGS	*Cryptococcus neoformans* (73,023), *P. jirovecii* (796)	< 37.5	Serum for cryptococcal antigen: positive
47	Not done	Sputum	tNGS	*P. aeruginosa* (6493), *H. haemolyticus* (80), adenovirus C (12), *C. albicans* (102), *P. jirovecii* (62), EBV (23), HHV-7 (4)	< 37.5	BAL for *M. tuberculosis* complex tNGS: 11 reads
48	Not done	BAL	tNGS	*K. pneumoniae* (56,021), *P. aeruginosa* (18,635), *S. aureus* (2661), *P. jirovecii* (1108), EBV (792)	Not done	BAL culture: *K. pneumoniae*, *P. aeruginosa*
49	Not done	Sputum	tNGS	*K. pneumoniae* (37,575), *S. aureus* (13,401), *P. aeruginosa* (13,123), *H. haemolyticus* (25), *C. albicans* (6927), *P. jirovecii* (201)	< 37.5	None
50	Not done	BAL	tNGS	*K. pneumoniae* (73,975), *P. jirovecii* (56), CMV (12)	< 37.5	BAL culture: *K. pneumoniae*
51	Not done	Sputum	tNGS	*H. haemolyticus* (25), *T. whipplei* (1373), *P. jirovecii* (202)	Not done	Throat swab for SARS-CoV-2 RNA: positive
52	Negative	BAL	mNGS	*K. pneumoniae* (177), *P. jirovecii* (11), HHV-6B (1), *Corynebacterium propinquum* (442,826), *Dolosigranulum pigrum* (140,682), *V. parvula* (76,864), *C. acnes* (39,845), *Prevotella salivae* (32,519), *A. dentalis* (2687), *Megasphaera micronuciformis* (6162), *Moraxella nonliquefaciens* (3916), *C. concisus* (4279), *S. epidermidis* (2934), *Streptococcus anginosus* (855), *S. salivarius* (588), *C. albicans* (5555)	< 37.5	None
53	Negative	BAL	tNGS	*P. jirovecii* (27)	< 37.5	None

BAL, Bronchoalveolar lavage; NGS, Next-generation sequencing; tNGS, Targeted NGS; mNGS, Metagenomics NGS; EBV, Epstein-Barr virus; CMV, Cytomegalovirus; HHV, Human herpes virus; HSV, Herpes simplex virus; AFB, Anti-fast bacilli; NPS, Nasopharyngeal swab; SARS-CoV-2, Severe acute respiratory syndrome coronavirus 2

### Other Microbiology Tests

Serum cryptococcal antigen was positive in one patient (case 46), and acid-fast bacilli were detected in two patients (cases 20 and 39) and *M. tuberculosis* DNA in one patient (case 39) (Table 2). HSV DNA was positive in the BAL of one patient (case 43). Influenza A virus and SARS-CoV-2 RNA were each positive in the nasopharyngeal swab of two patients respectively (cases 23 and 51) (Table 2).

### *P. jirovecii* Infection and Colonization

According to our case definitions, 37 (69.8%) of the 53 patients with *P. jirovecii* sequence reads detected in their respiratory samples had *P. jirovecii* infection, whereas the other 16 (30.2%) of the 53 were considered as colonization. *P. jirovecii* infection was associated with the presence of underlying disease with immunosuppression (35/37, 94.6%) compared to *P. jirovecii* colonization (3/16, 18.8%) (*P* < 0.05) (Table 3). Only two patients with *P. jirovecii* pneumonia did not have major immunosuppression. The first one (case 21, Table 1) was a 71-year-old man with membranous nephropathy, hypertension, diabetes mellitus and coronary heart disease. He refused to receive corticosteroid and other immunosuppressive treatment for his membranous glomerulonephritis. The second one (case 44, Table 1) was a 73-year-old man with chronic obstructive pulmonary disease, coronary heart disease, gout, hypertension, renal calculi and benign prostatic hyperplasia. In addition to underlying diseases, patients with *P. jirovecii* infection were associated with higher number of *P. jirovecii* sequence reads in their respiratory samples than those with *P. jirovecii* colonization (*P* < 0.005) (Table 3). Furthermore, there were significantly more patients with positive serum 1,3-β-D-glucan results in the infection (14/34, 41.2%) than in the colonization group (0/11) (*P* < 0.01) (Table 3). On the other hand, *P. jirovecii* colonization was associated with a predominance of the male sex (15/16, 93.8%) compared to *P. jirovecii* infection (20/37, 54.1%) (*P* < 0.01) (Table 3). Patients with *P. jirovecii* colonization was also associated with another definitive infectious disease diagnosis of the respiratory tract (7/16, 43.8%) compared to *P. jirovecii* infection (1/37) (*P* < 0.001) (Table 3). The definitive diagnoses of these seven patients were psittacosis (case 8), *M. kansasii* pulmonary infection (case 20), cryptococcosis (case 46), tuberculosis (case 39 and 47), influenza (case 23) and COVID-19 (case 51) (Table 2). Moreover, a significantly higher proportion of patients with *P. jirovecii* colonization (by definition did not receive specific anti-*P. jirovecii* treatment, 16/16, 100%) had improved compared to those with *P. jirovecii* infection (by definition received specific anti-*P. jirovecii* treatment, 25/37, 67.6%) (*P* < 0.01) (Table 3).

**Table 3:** Comparison of characteristics in patients with *Pneumocystis jirovecii* infection and colonization

Patient characteristics		Infection (*n* = 37)	Colonization (*n* = 16)	*P*-value
Age (years)		57.5 ± 12.7	63.4 ± 13.7	0.1356
Sex	Female	17	1	0.0051
	Male	20	15	
Underlying immunocompromised condition	All	35	3	< 0.0001
	HIV	3	0	
	Solid tumour on chemotherapy	18	0	
	Haematological malignancy	3	0	
	Connective tissue disease/autoimmune disease	14	2	
	Solid organ transplant	3	1	
Clinical manifestations	Fever	19	11	0.2407
	Cough	15	12	0.0212
	Shortness of breath	24	6	0.065
Laboratory test	^a^Median number of *P. jirovecii* sequence reads detected via tNGS in 44 of 53 patients (interquartile range), /100 K original reads	236 (108, 8319)	52.50 (11.5, 193)	0.002
	Positive GMS staining respiratory samples in 31 of 53 patients	6/27	0/4	0.5614
	Positive serum 1,3-β-D-glucan in 45 of 53 patients	14/34	0/11	0.0098
	Definitive diagnosis of other infectious diseases	1	7	0.0005
Outcome	Improved	25	16	0.0096
	Succumbed	12	0	

tNGS, Targeted next-generation sequencing; GMS, Gomori methenamine silver

^a^All the other nine patients with *P. jirovecii* detected via metagenomics NGS had *P. jirovecii* infection

## Discussion

In this study, 53 patients in our hospital with *P. jirovecii* sequences in their respiratory samples were detected by tNGS or mNGS analysis. Among these 53 patients, only three were HIV-positive (cases 12, 41 and 45, Table 1), whereas the other 50 were HIV-negative. This is very different from the general epidemiology of *P. jirovecii* infections, of which HIV infection is the single most important risk factor. The common reasons for immunosuppression in the HIV-negative patients in this cohort were solid tumour or haematological malignancies on chemotherapy and autoimmune diseases or solid organ transplant recipients on corticosteroid and/or other immunosuppressive treatment, which is consistent with the changing epidemiological profile of *P. jirovecii* infection in the past decades [22]. For HIV-positive patients with *P. jirovecii* infections, the fungal loads in their respiratory tracts are usually high and direct microscopic examination after GMS staining, sometimes even using induced sputum samples, is often sufficient for making a diagnosis. In contrast, for the other immunocompromised patients, the fungal load is usually low and bronchoscopic examination has to be performed to collect BAL samples so as to improve the yield. In fact, for all the three HIV-positive patients in the present cohort, their BAL samples were also positive for *P. jirovecii* by direct microscopic examination after GMS staining, whereas for the 29 BAL samples obtained from the HIV-negative patients that were submitted for microscopic examination after GMS staining, only three were positive for *P. jirovecii* (*P* < 0.001 by Fisher’s Exact test). This is in line with the high number of *P. jirovecii* sequence reads (84,000 for cases 12, 58,385 for case 41 and 64,629 for case 45) (Table 2) observed in the three BAL samples collected from the three HIV-positive patients examined by tNGS, which is significantly higher than the number of *P. jirovecii* sequence reads (median 655, range 27 to 48,609) in the BAL samples collected from the 21 HIV-negative patients examined by tNGS (*P* < 0.001 by Mann–Whitney U test).

Detection of *P. jirovecii* sequence reads in respiratory samples has to be interpreted discreetly. Traditionally, *P. jirovecii* infection was diagnosed in the laboratory by direct detection of *P. jirovecii* asci in respiratory samples after GMS staining in immunocompromised patients with suspected clinico-radiological features, such as shortness of breath, hypoxia and ground glass infiltrates on chest radiographs. In the past decades, a number of PCR assays have been developed for the detection of *P. jirovecii* in respiratory tract specimens [3–6]. In some of these studies, colonization of *P. jirovecii* in the respiratory tract has been suggested [5–8]. In the present cohort, 16 (29.6%) of the 53 patients with *P. jirovecii* sequences in their respiratory samples detected by NGS analysis recovered without receiving specific anti-*P. jirovecii* therapy. In some of these 16 patients, other respiratory pathogens were present. For example, *C. psittaci* was detected from the sputum of Case 8 and he responded to doxycycline well; and in Cases 23 and 51, influenza A virus and SARS-CoV-2 RNA were detected in their nasopharyngeal and throat swabs respectively (Table 2). All of the 16 patients improved after receiving specific antimicrobial therapy to the other pathogens identified or just symptomatic treatment. In these 16 patients, *P. jirovecii* was considered as colonizers of the respiratory tract, rather than pathogens; and they were associated with the male gender, absence of underlying disease, negative serum 1,3-β-D-glucan, and a lower number of *P. jirovecii* sequence reads (Table 3). It is interesting to note that in our recent study on the detection of *T. whipplei* in respiratory samples by NGS, Whipple disease was never suspected to be a diagnosis in any of the patients before detection of the bacterium; and the presence of *T. whipplei* in the respiratory specimens of these patients was still elusive [21].

**Fig. 1:**
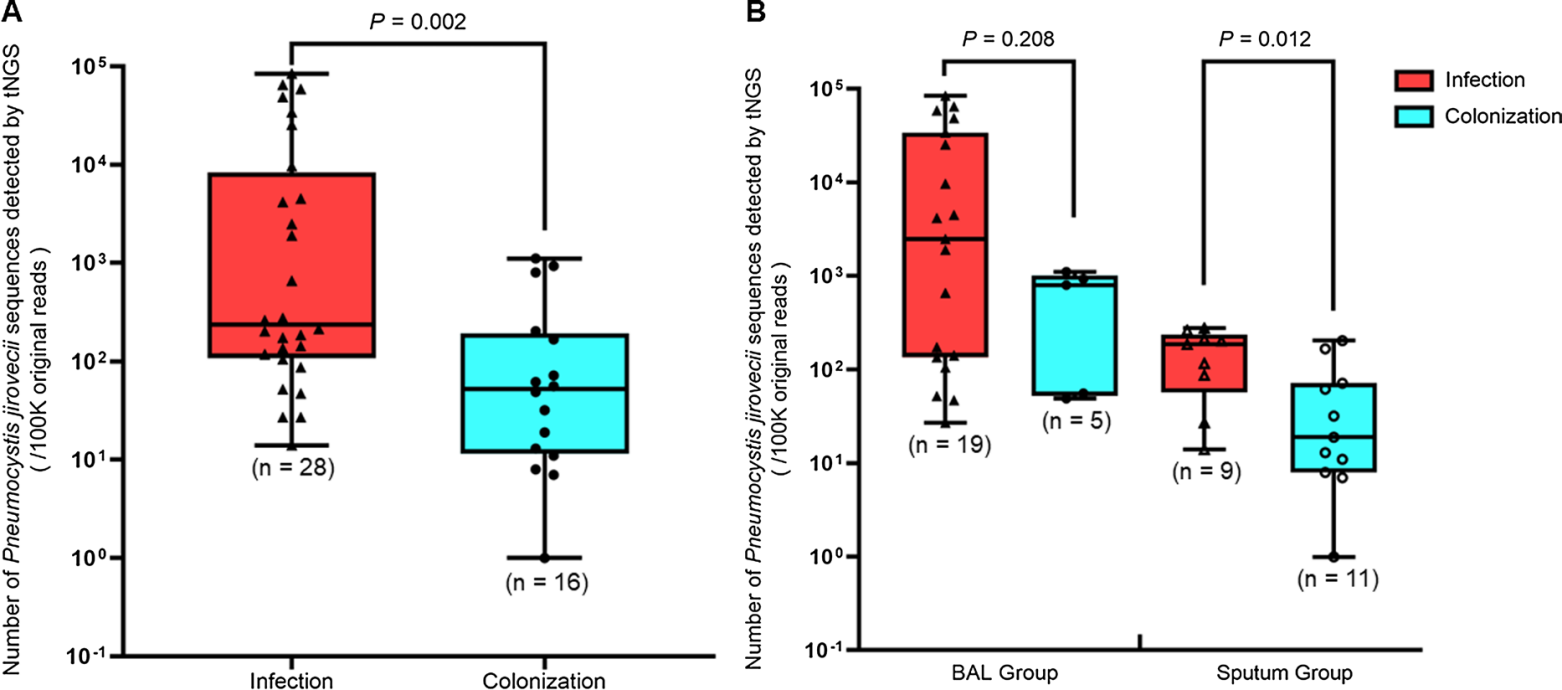
Distribution in number of *P. jirovecii* sequence reads in respiratory samples from patients in the present cohort detected by targeted next-generation sequencing (tNGS). Panel A: boxplot showing number of *P. jirovecii* sequence reads distribution in all respiratory samples from patients with *P. jirovecii* infection and colonization. Panel B: boxplot showing number of *P. jirovecii* sequence reads distribution in bronchoalveolar lavage (BAL) and sputum samples from patients with *P. jirovecii* infection and colonization

Although infection is associated with a significantly higher number of *P. jirovecii* sequence reads as compared to colonization, clinical judgement is still the most crucial in determining whether a particular case is genuine *P. jirovecii* pneumonia. When the number of *P. jirovecii* sequence reads in all respiratory (sputum and BAL) samples between the infection and colonization groups were compared, it was observed that the number of sequence reads was significantly higher in the infection than the colonization group (*P* < 0.005) (Fig. 1A). However, for example, if 79.5 reads were used as the cutoff for distinguishing between *P. jirovecii* pneumonia and colonization, only a sensitivity of 82.14% and a specificity of 68.75% could be achieved. Furthermore, when the analysis was performed for the sputum group, the number of sequence reads was still significantly higher in the infection than the colonization group (*P* < 0.05); but when the analysis was performed for the BAL group, there was no difference between the number of reads in the two groups, although there was still a trend towards a higher number of reads in the infection than the colonization group (Fig. 1B). All these showed that the number of sequence reads is not a reliable parameter to indicate whether a particular patient has *P. jirovecii* pneumonia or just *P. jirovecii* colonization. In contrast to the number of *P. jirovecii* sequence reads, it was shown in the present cohort that positive serum 1,3-β-D-glucan and direct GMS staining of respiratory samples were highly specific, although not sensitive, for *P. jirovecii* infection (Table 3); and hence would be useful for the prediction of *P. jirovecii* pneumonia if these results were positive. The final diagnosis of *P. jirovecii* pneumonia should be made using a combination of clinical, radiological and laboratory findings.

### Supplementary Information

Below is the link to the electronic supplementary material.

Supplementary file1 (DOCX 17 KB)

## References

[1] Morris A, Norris KA (2012). Colonization by *P. jirovecii* and its role in disease. Clin Microbiol Rev.

[2] Desoubeaux G, Franck-Martel C, Caille A, Drillaud N, Carluer L, de Kyvon MA, Bailly É, Chandenier J (2017). Use of calcofluor-blue brightener for the diagnosis of *P. jirovecii* pneumonia in bronchial-alveolar lavage fluids: a single-center prospective study. Med Mycol.

[3] Wakefield AE, Pixley FJ, Banerji S, Sinclair K, Miller RF, Moxon ER, Hopkin JM (1990). Detection of Pneumocystis carinii with DNA amplification. Lancet.

[4] Robberts FJ, Liebowitz LD, Chalkley LJ (2007). Polymerase chain reaction detection of Pneumocystis jiroveci: evaluation of 9 assays. Diagn Microbiol Infect Dis.

[5] Huggett JF, Taylor MS, Kocjan G, Evans HE, Morris-Jones S, Gant V, Novak T, Costello AM, Zumla A, Miller RF (2008). Development and evaluation of a real-time PCR assay for detection of *P. jirovecii* DNA in bronchoalveolar lavage fluid of HIV-infected patients. Thorax.

[6] To KK, Wong SC, Xu T, Poon RW, Mok KY, Chan JF, Cheng VC, Chan KH, Hung IF, Yuen KY (2013). Use of nasopharyngeal aspirate for diagnosis of pneumocystis pneumonia. J Clin Microbiol.

[7] Aguilar YA, Rueda ZV, Maya MA, Vera C, Rodiño J, Muskus C, Vélez LA (2021). Is it possible to differentiate *P. jirovecii* pneumonia and colonization in the immunocompromised patients with pneumonia?. J Fungi.

[8] Fan LC, Lu HW, Cheng KB, Li HP, Xu JF (2013). Evaluation of PCR in bronchoalveolar lavage fluid for diagnosis of *P. jirovecii* pneumonia: a bivariate meta-analysis and systematic review. PLoS ONE.

[9] Wilson MR, Naccache SN, Samayoa E, Biagtan M, Bashir H, Yu G, Salamat SM, Somasekar S, Federman S, Miller S, Sokolic R, Garabedian E, Candotti F, Buckley RH, Reed KD, Meyer TL, Seroogy CM, Galloway R, Henderson SL, Gern JE, DeRisi JL, Chiu CY (2014). Actionable diagnosis of neuroleptospirosis by next-generation sequencing. N Engl J Med.

[10] Tsang CC, Teng JLL, Lau SKP, Woo PCY (2021). Rapid genomic diagnosis of fungal infections in the age of next-generation sequencing. J Fungi.

[11] Xing F, Hung DLL, Lo SKF, Chen S, Lau SKP, Woo PCY (2022). Next-generation sequencing-based diagnosis of bacteremic Listeria monocytogenes meningitis in a patient with anti-interferon gamma autoantibodies: a case report. Infect Microb Dis.

[12] Xing F, Ye H, Deng C, Sun L, Yuan Y, Lu Q, Yang J, Lo SKF, Zhang R, Chen JHK, Chan JFW, Lau SKP, Woo PCY (2022). Diverse and atypical manifestations of Q fever in a metropolitan city hospital: Emerging role of next-generation sequencing for laboratory diagnosis of Coxiella burnetii. PLoS Negl Trop Dis.

[13] Xing F, Yang Q, Deng C, Sun L, Luo Z, Ye H, Yang J, Lo SKF, Lau SKP, Woo PCY (2022). Clinical impact of next-generation sequencing on laboratory diagnosis of suspected culture-negative meningitis and encephalitis. J Infect.

[14] Jiang J, Bai L, Yang W, Peng W, An J, Wu Y, Pan P, Li Y (2021). Metagenomic next-generation sequencing for the diagnosis of *P. jirovecii* pneumonia in Non-HIV-infected patients: a retrospective study. Infect Dis Ther.

[15] Wang D, Fang S, Hu X, Xu Q, Chu X, Mei X, Xie W (2022). Metagenomic Next-Generation Sequencing Is Highly Efficient in Diagnosing *P. jirovecii* Pneumonia in the Immunocompromised Patients. Front Microbiol.

[16] Lu X, Zhang J, Ma W, Xing L, Ning H, Yao M (2022). Pneumocystis Jirovecii pneumonia diagnosis via metagenomic next-generation sequencing. Front Med.

[17] Donnelly JP, Chen SC, Kauffman CA, Steinbach WJ, Baddley JW, Verweij PE, Clancy CJ, Wingard JR, Lockhart SR, Groll AH, Sorrell TC, Bassetti M, Akan H, Alexander BD, Andes D, Azoulay E, Bialek R, Bradsher RW, Bretagne S, Calandra T, Caliendo AM, Castagnola E, Cruciani M, Cuenca-Estrella M, Decker CF, Desai SR, Fisher B, Harrison T, Heussel CP, Jensen HE, Kibbler CC, Kontoyiannis DP, Kullberg BJ, Lagrou K, Lamoth F, Lehrnbecher T, Loeffler J, Lortholary O, Maertens J, Marchetti O, Marr KA, Masur H, Meis JF, Morrisey CO, Nucci M, Ostrosky-Zeichner L, Pagano L, Patterson TF, Perfect JR, Racil Z, Roilides E, Ruhnke M, Prokop CS, Shoham S, Slavin MA, Stevens DA, Thompson GR, Vazquez JA, Viscoli C, Walsh TJ, Warris A, Wheat LJ, White PL, Zaoutis TE, Pappas PG (2020). Revision and update of the consensus definitions of invasive fungal disease from the European organization for research and treatment of cancer and the mycoses study group education and research consortium. Clin Infect Dis.

[18] Carroll KC, Pfaller MA (2019). Manual of clinical microbiology.

[19] Lau SK, Tang BS, Teng JL, Chan TM, Curreem SO, Fan RY, Ng RH, Chan JF, Yuen KY, Woo PC (2014). Matrix-assisted laser desorption ionisation time-of-flight mass spectrometry for identification of clinically significant bacteria that are difficult to identify in clinical laboratories. J Clin Pathol.

[20] Xing F, Xia Y, Lu Q, Lo SKF, Lau SKP, Woo PCY (2023). Rapid diagnosis of fatal Nocardia kroppenstedtii bacteremic pneumonia and empyema thoracis by next-generation sequencing: a case report. Front Med.

[21] Xing F, Lo SW, Liu M, Deng C, Ye H, Sun L, Yang J, Lo SKF, Lau SKP, Woo PCY (2023). Emergence of Tropheryma whipplei detection in respiratory samples by next-generation sequencing: pathogen or innocent bystander?. J Infect.

[22] Cillóniz C, Dominedò C, Álvarez-Martínez MJ, Moreno A, García F, Torres A, Miro JM (2019). *Pneumocystis* pneumonia in the twenty-first century: HIV-infected versus HIV-uninfected patients. Expert Rev Anti Infect Ther.

