# Supplementary material for: Rapid Diagnosis of Pneumocystis jirovecii Pneumonia and Respiratory Tract Colonization by Next-Generation Sequencing
Source: Mycopathologia. 2024 May 5;189(3):38. doi: 10.1007/s11046-024-00849-y (PMC11070382; doi:10.1007/s11046-024-00849-y)
Supplement: Supplementary file 1 — Supplementary file1 (DOCX 17 KB) [file 11046_2024_849_MOESM1_ESM.docx]

**Supplementary Table 1. Microorganisms considered as contaminants or colonizers of oral cavity detected in respiratory samples from 26 of the 53 patients by NGS**

| **Classification** | **Species** |
| --- | --- |
| Bacteria | *Abiotrophia defectiva*, *Actinomyces dentalis*, *Actinomyces graevenitzii*, *Actinomyces oris*, *Aggregatibacter segnis*, *Atopobium parvulum*, *Campylobacter concisus*, *Campylobacter curvus*, *Capnocytophaga granulosa*, *Capnocytophaga sputigena*, *Corynebacterium matruchotii*, *Corynebacterium propinquum*, *Corynebacterium simulans*, *Corynebacterium striatum*, *Corynebacterium tuberculostearicum*, *Cryptobacterium curtum*, *Dolosigranulum pigrum*, *Eikenella corrodens*, *Filifactor alocis*, *Fusobacterium nucleatum*, *Gemella haemolysans*, *Gemella sanguinis*, *Granulicatella adiacens*, *Haemophilus haemolyticus*, *Haemophilus parainfluenzae*, *Leptotrichia buccalis*, *Megasphaera micronuciformis*, *Mogibacterium timidum*, *Moraxella nonliquefaciens*, *Neisseria bacilliformis*, *Neisseria flavescens*, *Olsenella uli*, *Oribacterium sinus*, *Parvimonas micra*, *Peptoniphilus lacrimalis*, *Peptostreptococcus anaerobius*, *Peptostreptococcus stomatis*, *Prevotella denticola*, *Prevotella melaninogenica*, *Prevotella salivae*, *Prevotella veroralis*, *Rothia mucilaginosa*, *Solobacterium moorei*, *Streptococcus anginosus*, *Streptococcus infantis*, *Streptococcus mitis*, *Streptococcus oralis*, *Streptococcus parasanguinis*, *Streptococcus pseudopneumoniae*, *Streptococcus salivarius*, *Treponema denticola*, *Veillonella dispar*, *Veillonella parvula* |
| Yeasts | *Candida albicans*, *Candida tropicalis* |

Abbreviation: NGS, next-generation sequencing
